# Data of the MAternal Mood Assessment (MAMA) survey for healthcare professionals: A pilot study on midwives in Italy

**DOI:** 10.1016/j.dib.2023.109902

**Published:** 2023-12-02

**Authors:** Claudia Ravaldi, Alfredo Vannacci

**Affiliations:** PEARL Perinatal Research Laboratory, CiaoLapo Foundation, Department of Neurosciences, Psychology, Drug Research and Child Health, University of Florence, Viale Pieraccini 6, 50139 Florence, Italy

**Keywords:** Perinatal depression, Maternal mental health, Healthcare professionals, Risk factors, Midwifery

## Abstract

This article describes the data collected from a survey of 152 midwives in Italy on their knowledge, attitudes, and practices regarding perinatal depression. The survey used the MAternal Mood Assessment (MAMA) questionnaire, a 35-item tool that covers various aspects of perinatal depression, such as definition, prevalence, risk factors, diagnosis, management, and support. The data provides valuable insights into the training needs and experiences of midwives in the area of maternal mental health, which can inform the development of interventions and education programs. The full dataset is available in Mendeley Data repository.

Specifications Table

**Table:** 

Subject	Psychiatry and Mental Health
Specific subject area	Maternal Mental Health, in particular perinatal depression, and midwifery care
Data format	Raw, Analyzed
Type of data	Table, Figure
Data collection	The data were collected using the MAternal Mood Assessment (MAMA) questionnaire, which is a self-administered tool that evaluates midwives' knowledge, attitudes, and practices regarding perinatal depression. The questionnaire consists of 37 items derived from previous literature and expert consultation. The questionnaire was administered online to a sample of 152 midwives working in different settings in Italy.
Data source location	PEARL Perinatal Research Laboratory, CiaoLapo Foundation, Department of Neurosciences, Psychology, Drug Research and Child Health, University of Florence, Viale Pieraccini 6, 50,139, Florence, Italy
Data accessibility	Repository name: Mendeley Data identification number: 10.17632/sx23zmtcxv.1 Direct URL to data: https://data.mendeley.com/datasets/sx23zmtcxv Instructions for accessing these data: freely available

## 1 Value of the Data

• Training Needs and Care Quality: The MAMA survey highlights areas where midwives require enhanced training in maternal mental health. This data can refine training approaches and ultimately benefit mothers and infants.
• Introduction of the MAMA Survey: We've presented the MAMA survey, a novel instrument to identify and address training gaps in maternal mental health among midwives.
• Resource for Research: The data from the MAMA survey is valuable for researchers across maternal mental health, midwifery, and medical education, facilitating new studies, improved training initiatives, and policy recommendations.

## 2 Background

Perinatal depression (PND) is a global health concern that affects roughly 12% of women worldwide during pregnancy and the postpartum period [1]. The prevalence varies significantly across countries with different income levels. Recent research indicates that in Italy, about 6.4% of women experience PND during pregnancy, and nearly 20% experience it during the postpartum period [2]. Despite its high occurrence, there is a consensus among healthcare professionals that PND is often not diagnosed [3]. The repercussions of PND are severe, affecting both the mother and the child, from attachment issues to emotional and cognitive development impairments. It's also crucial to note the heightened risk of suicide during the postpartum period due to PND, which is one of the leading causes of maternal mortality in the first year after birth in high-income countries, including Italy [4].

Midwives have a unique and crucial role in the lives of childbearing women, making them instrumental in identifying mental health issues in this group. However, the lack of a specific tool for PND and the challenge of differentiating depressive symptoms from typical physical and mental characteristics of the pre and post-partum periods must be taken into account. While routine PND screening for all women is recommended in the literature, there is no agreement on the most effective tool. One approach could be to identify risk factors during prenatal check-ups, although these factors are not reliable predictors of depressive disorders. Therefore, if midwives suspect PND, they should refer the woman to a mental health professional for a formal evaluation.

Understanding the problem and personal attitudes towards it, which can lead to prejudiced emotional reactions, are two components of stigma [5,6].

Given these factors and the role midwives play in the mental health of childbearing women, we promoted the “MAternal Mood Assessment” (MAMA) study to evaluate their knowledge and perceptions of PND.

## 3 Data Description

The MAMA survey provides a rich source of information about the perspectives and experiences of healthcare professionals in dealing with perinatal depression, which can be valuable for informing interventions and training.

**Fig. 1:**
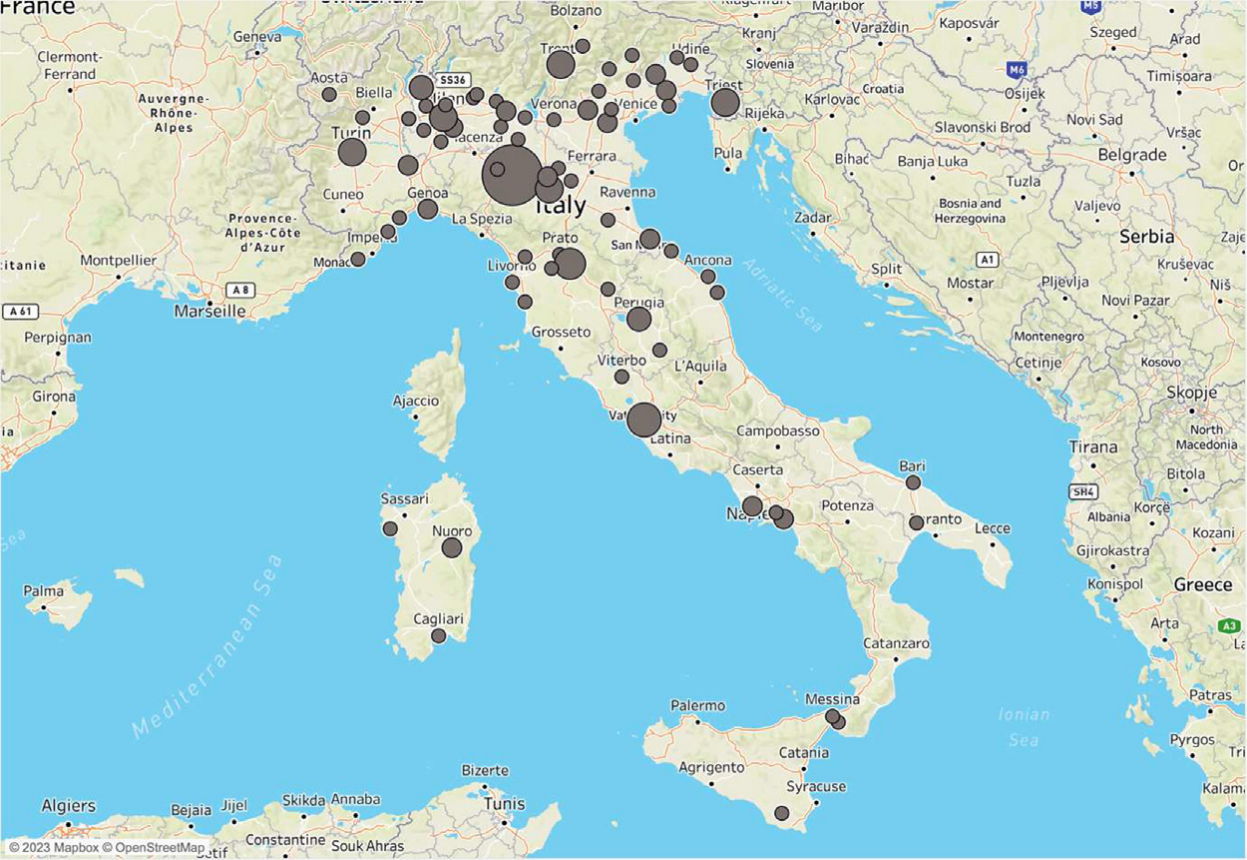
Geographical distribution of the sample.

Table 1 describes the characteristics of the sample. Participating midwives are fairly evenly distributed across three age groups, ranging from early twenties to late fifties. Their work experience varies from less than a year to over three decades. The majority of participants are located in the North of Italy and work in a hospital setting. The specific workplaces within the healthcare system are diverse, including birthing rooms, obstetrics wards, and private practices, among others. Fig. 1 shows the geographical distribution of the sample.

**Table 1:** General characteristics of the sample.

	Summary
*N*	152
Age classes	
21.0–24.9	53 (34.9%)
26.0–30.9	51 (33.6%)
32.0–58.9	48 (31.6%)
Work years classes	
0.0–0.9	59 (38.8%)
2.0–2.9	26 (17.1%)
4.0–8.9	29 (19.1%)
10.0–34.9	38 (25.0%)
Italian geographical zones	
Center	28 (18.4%)
North	109 (71.7%)
South	15 (9.9%)
Works in hospital	
No	53 (34.9%)
Yes	99 (65.1%)
Place of work	
University	14 (9.2%)
Birthing room	80 (52.6%)
Obstetrics ward	78 (51.3%)
Pregnancy pathology ward	30 (19.7%)
Midwifery outpatient clinic	33 (21.7%)
Territorial services	14 (9.2%)
Private practice	13 (8.6%)

Table 2 describes professional experiences of the sample. A majority of the participants reported that there are no protocols for post-natal depression in their workplaces. Most of them have not attended courses on perinatal mental health. When it comes to assisting patients with post-natal depression, a significant portion of the participants have either never assisted such patients or have assisted fewer than five. A smaller number of participants have assisted between 5 and 10 patients, and only a few have assisted more than 10 patients.

**Table 2:** Professional experiences of the sample.

	Summary
N	152
In your working place are there protocols for post-natal depression?	
No	103 (67.8%)
Yes	49 (32.2%)
Have you ever attended courses on perinatal mental health?	
No	115 (75.7%)
Yes	37 (24.3%)
Number of patients with post-natal depression assisted	
None	50 (32.9%)
<5	73 (48.0%)
5–10	21 (13.8%)
>10	8 (5.3%)

## 4 Experimental Design, Materials and Methods

The study was designed as a cross-sectional survey, with the primary aim of assessing the perceptions and experiences of healthcare professionals regarding maternal mood disorders during the perinatal period.

MAMA is a comprehensive survey tool designed for healthcare professionals to assess their knowledge, attitudes, and experiences regarding perinatal depression. The questionnaire, made up of 37 questions that are either multiple-choice or open-ended, was crafted by blending symptoms from the DSM-5 and crucial risk factors found in medical studies. Extra questions were added by the authors to better gauge the midwives' understanding of perinatal depression.

The MAMA questionnaire uses a variety of response formats, including multiple-choice questions, Likert scale ratings, and open-ended questions. The Likert scale ratings range from 0 (not at all) to 4 (very much). No composite score was calculated and no item was reversed. Some open-ended questions allow respondents to provide more detailed and nuanced responses.

The survey is divided into several sections, each focusing on a different aspect of perinatal depression.

The first section (A) collects socio-demographic information about the healthcare professionals, including their age, gender, professional qualification, year of graduation, years of service, and the location and context of their current work.

The second section (B) focuses on the healthcare professionals' training and experience in dealing with perinatal depression. This includes questions about whether there is a specific pathway for perinatal depression in their facility, whether they have participated in specific training on perinatal depression, and how often they have encountered mothers with a diagnosis of perinatal depression.

The third section (C) of the survey aims to assess the healthcare professionals' understanding and perception of perinatal depression. This includes questions about the definition of perinatal depression, the perceived prevalence, risk factors, and the most appropriate reactions to maintain a good relationship with a mother showing symptoms of perinatal depression.

The fourth section (D) focuses on the healthcare professionals’ experiences and feelings when dealing with cases of perinatal depression. This includes questions about their reactions upon learning that a patient is suffering from perinatal depression, their interactions with the family, and their opinions on the most effective approaches to support women with symptoms of perinatal depression.

The fifth section (E) of the survey asks about the healthcare professionals’ opinions on various aspects of managing perinatal depression, such as breastfeeding, the role of the father, the involvement of different professional figures, and the usefulness of having guidelines on the management of perinatal depression.

A final section (F) is comprised of three open-end questions, asking free-text responders’ opinions on the most important and the most difficult aspects in the management of perinatal depressive disorders, and a textbox for any free comment.

The structure of the survey is reported in Table 3

**Table 3:** Structure of MAMA survey.

No.	Question	Response options	Values
**Section A -** socio-demographic information			
1	Age	Open-ended	n
2	Gender	Male, Female	1,2
3	Qualification and profession	Doctor, Midwife, Nurse, Psychologist, Other (specify)	1–5
4	Year of graduation or equivalent qualification	Open-ended	N
5	Please indicate the university, institute or city where you obtained your degree	Open-ended	Text
6	Years of work service	Open-ended	n
7	Where do you currently work?	Region, City	Text
8	In which professional context do you work?	*Multiple choice possible* University Clinic, Birthing Room, Obstetric Ward, Pregnancy Pathology, Obstetric Clinic, Counseling, Private Practice, Currently Not Working, Other (specify)	0,1
**Section B -** training and experience			
9	In your facility, is there a specific pathway for perinatal depression?	No, Yes, I don't know	0,1,2
10	Have you ever attended a specific training course on perinatal depression?	No, Yes, I don't remember	0,1,2
11	Have you ever assisted a mother with a diagnosis of perinatal depression during your career? If yes, how many times?	Never, <5 times, 5–10 times, >10 times	0–3
**Section C -** understanding and perceptions			
12	Which of the following do you think could be the best definition of perinatal depression?	Presence of a depressive disorder during pregnancy, Presence of a depressive disorder during pregnancy or after birth, Presence of a depressive disorder in childbearing age, Other (specify)	1–4
13	In your opinion, how many women out of a hundred pregnancies are affected annually by perinatal depression?	Open-ended	n
14	In your opinion, how important can the following possible risk factors be considered for the development of depression during pregnancy or after birth?	List of 23 risk factors each one graded as: Not at all, a little, enough, a lot, very much Primiparity, Age <20y, Age >35y, Previous losses, Familiar issues, Social isolation, Hostility, Previous anxiety, Perfectionism, Low self-esteem, Couple difficulties, Previous depression, Familiar depression, Social expectations, Difficult newborn, Issues with one's mother, History of sexual abuse, Unwanted pregnancy, Trauma during pregnancy, Diabetes, Gestational diabetes, Pregnancy complications, Single mom	0–4
15	In your experience, in which phase of pregnancy do depressive symptoms most frequently occur?	In the first trimester, In the second trimester, In the third trimester, I don't know	1–4
16	Based on your experience regarding women's requests for help, would you say that women with depression during pregnancy...	*Multiple choice possible* Do not ask for help, Ask for help during childbirth preparation courses, Ask for help during routine interviews with the caregiver, Turn to peer groups (non-healthcare), I don't know	0,1
17	In your opinion, how useful can the following intervention methods be in case of depression during pregnancy?	List of 10 interventions, each graded as: Not at all, a little, enough, a lot, very much Epidural analgesia - Induction of labor - Cesarean delivery - Sedation during childbirth – Abortion - Use of antidepressants – Counseling – Psychoeducation - Sleep hygiene - Cognitive-behavioral therapy	0–4
**Section D -** experiences and feelings			
18	In your experience, in which period do depressive symptoms post-partum most frequently occur?	In the first 24 h, In the first week, After two weeks, Within a year	1–4
19	How often in your working career have you encountered women who during the puerperium presented one or more of the following signs or symptoms?	List of 47 symptoms, each graded as: Never, Almost never (<5%), Rarely (5–10%), Not very frequently (10–20%), Quite frequently (>20%) Prolonged asthenia - Sadness without cause - Episodes of repeated crying - Continual irritability - Difficulty falling asleep - Loss of appetite/anorexia - Constant weight loss / constant weight gain - Generalized anxiety - Depressed mood - Loss of interest in daily activities - Sense of danger/alert for the child - Disinterest in the child - Aggressiveness towards the child - Insomnia - Hypersomnia - Motor slowing down - Fear of not being fit to be a mother - Belief of being an incapable mother - Feeling guilty - Fear of not interpreting the newborn's cry correctly - Difficulty in tolerating the crying of the newborn - Repeated difficulty falling asleep - Repeated difficulty falling back asleep after waking up - Difficulty in taking care of the child - Difficulty in taking care of one's own home environment - Difficulty taking care of oneself - Difficulty asking for help - Overcommitment - Loss of interest in the newborn - Auditory hallucinations - Presence of delusions - Lack of insight - Belief of being in danger due to the presence of the child - Belief of being in danger due to operators or family members - Indifference to the needs of the newborn - Physical agitation - Indecision and worry - Suicidal ideation - Fear of going down the stairs with the baby - Fear of leaning out the window with the baby in your arms - Fear of handling knives in the same room as the child - Having attempted suicide - Feeling like a failure - Negative thoughts about the future - Recurring thoughts of death - Difficulty leaving the house with the child - Difficulty concentrating	0–4
20	What feelings have you experienced during the interview with a woman who presents symptoms of perinatal depression?	Embarrassment, Fear of saying the wrong things, Fear of doing the wrong things, Annoyance, Anger, Detachment, Disbelief (how can she be sad when everything went	0,1
21	As a professional, how did you experience the information that one of your patients suffers from perinatal depression?	It has never happened to me, Bad (I feel I underestimated it), Inadequate, Normal (part of my duties), I identified with her, Other (please specify)	0,1
**Section E -** opinions on management			
22	Among the reactions listed, which do you think are the most appropriate to maintain a good relationship with a mother who presents symptoms of perinatal depression?	Show respect for her condition, Show empathy, Stand by her, Listen to her needs, Show interest and attention to her problem, Offer availability to talk about it, Encourage her by listing all the things that have gone well, Motivate her to react for the good of the child, Entrust her to a more experienced colleague and stop seeing her, Send her immediately to the psychologist, Offer continuity of care, I don't know, Other (specify)	0,1
23	In your opinion, regarding the role of the father in maternal depression:	An empathetic father can be a great support, A father has a higher risk of experiencing depressive symptoms if his partner has a depressive disorder, Fathers never suffer from postnatal depression, Fathers must take care of the child instead of the mother, A father must encourage the mother to feel better, I don't know, Other (specify)	0,1
24	In your opinion, when dealing with a diagnostic suspicion of perinatal depression, it is more appropriate to:	Wait to see how it evolves, Immediately notify the psychiatry colleagues, Personally talk to the reference psychologist, First of all, talk to the woman's family members	0–3
25	In your opinion, when dealing with a diagnostic suspicion of perinatal depression what is more effective to establish an alliance with the woman:	Use open-end questions, Use close questions, Give a questionnaire, Give an informative flyer about depression, Don't know	0,1
26	In your opinion, when dealing with a diagnostic suspicion of perinatal depression, which are the best ways to interact with family members?	Communicate the diagnostic suspicion to them first to prepare them, Communicate first to the woman and then together with her decide which family members to discuss the problem with, Communicate to the family the contact of the psychiatrist so that they accompany the woman, Start a support path for the whole family in order to improve the outcomes, I don't know	0,1
27	Among these figures, with whom do you find it easier to talk about cases of perinatal depression that you assist?	Midwives, Gynecologists, Psychologists, Psychiatrists, My family or friends, It has never happened to me, Other (specify)	0,1
28	Which of the following approaches do you think could be useful to support women with symptoms of depression in pregnancy?	Schedule subsequent meetings after discharge, Think about a new pregnancy within six months at most, Create contact between the woman and the clinic, Personally call the woman, Inform the family of the feelings the woman may feel, Give information material on perinatal depression, Activate the home service, Make an appointment for a control visit in the ward, Activate the perinatal psychiatry service, Leave the woman free to do what she wants, Other (specify)	0,1
29	In your opinion, which professional figures should take care of offering support to the woman with postnatal depression?	Midwife, Psychologist, Gynecologist, Family doctor, Psychiatrist, Other (specify)	0,1
30	About breastfeeding in postnatal depression, how much do you agree with these statements?	It is contraindicated because the mother is stressed, It is possible if the mother wishes, It is always desirable, It is possible but only if mixed, It is useful because it keeps the mother busy, It is possible if the mother does not take drugs	1–3
31	Between your obstetric ward and the clinic, are there direct relationships on this topic?	No, Yes, I don't know	0,1,2
32	Does the clinic in your area offer an outpatient service for perinatal depression?	No, Yes, I don't know	0,1,2
33	In your area, is there a support network (associations, private entities under agreement…) for perinatal depression?	No, Yes (specify), I don't know	0,1,2
34	Would you find it useful to have guidelines on the management of perinatal depression?	No, Yes, I don't know	0,1,2
**Section F -** open-ended questions			
35	In your opinion, what are the three most important aspects in the management of perinatal depressive disorders?	Three separated open-ended answers	Text
36	In your opinion, what are the three most difficult aspects in the management of perinatal depressive disorders?	Three separated open-ended answers	Text
37	The survey is over. If you want to leave a comment of any kind, write it here	Open-ended	Text

The survey was administered to a sample of 152 midwives across Italy. The participants were selected using a convenience sampling method, with the questionnaire being distributed via email and social media platforms.

The data collected from the MAMA questionnaire was then analyzed using descriptive statistics to provide an overview of the healthcare professionals' perceptions and experiences of perinatal depression. This included calculating frequencies and percentages for the multiple-choice and Likert scale questions, and conducting a thematic analysis of the open-ended responses.

## Limitations

Not applicable

## Ethics Statement

The survey was voluntary and anonymous, no personal data were recorded, in no way it was possible to identify the single respondents. Informed consent was obtained from all participants. Data were acquired in compliance with GDPR regulation (General Data Protection Regulation, European Union 2016/679). When applicable, the research proposal was approved by participating hospital authorities.

## CRediT authorship contribution statement

**Claudia Ravaldi:** Conceptualization, Methodology, Investigation, Writing – original draft, Writing – review & editing. **Alfredo Vannacci:** Conceptualization, Methodology, Investigation, Writing – original draft, Writing – review & editing.

## Data Availability

MAMA v4 (Original data) (Mendeley Data)
